# Teachers’ Roles in Coping with School Violence from the Perspectives of Prospective Teachers: A Q Methodological Approach

**DOI:** 10.3390/bs14111099

**Published:** 2024-11-15

**Authors:** Taeeun Shim, Cheolhae Ye

**Affiliations:** 1 Department of Education, Dongguk University, Seoul 04620, Republic of Korea; shim2593@dongguk.edu; 2 Humanitas College, Kyung Hee University, Yongin 17104, Republic of Korea

**Keywords:** school violence, bullying, prospective teachers, Q methodology, mixed methods

## Abstract

This study explores prospective teachers’ perceptions of school violence and their role in addressing it. Using a mixed method called Q methodology, we quantitatively analyzed the subjective views of 37 prospective teachers. Based on 33 statements, the study categorizes teachers’ roles in managing school violence and analyzes the characteristics of each role type. The research results provide basic data for prospective teachers to develop their capabilities as experts in dealing with school violence. The study identifies the following three types of teachers: prevention-oriented (Type 1), reality-avoiding (Type 2), and legal-regulation-oriented (Type 3). Type 1 emphasizes that teachers can significantly prevent school violence and believes that trusting relationships between teachers and students are key to reducing school violence. Type 2 teachers tend to ignore school violence out of fear of harm, even though they acknowledge its seriousness. Type 3 emphasizes the strict enforcement of legal responsibilities and rules, believing that clear regulations and punishments are essential for reducing school violence. This study highlights the need for customized educational programs that reflect the characteristics of different teacher types in handling school violence. It suggests incorporating practical strategies for preventing and responding to school violence in teacher training, expanding mental-health education, and promoting cooperative conflict-resolution methods between students and teachers.

## 1. Introduction

School violence is a serious social problem worldwide, including in the United States, Europe, Asia, the Middle East, and Africa [1,2]. Recently, in Korea, interest in school violence has increased again as dramas about victims of school violence in high school becoming adults and taking revenge on the perpetrators have become very popular. In addition, national interest has grown even more as news reports show children of high-ranking public officials, celebrities, and athletes involved in school violence [3,4].

Previous school-violence studies have mainly focused on violence between students. However, recently, there has been awareness that teachers can also be perpetrators and victims of violence from students, parents, and fellow teachers. This situation suggests that schools need to deal with violence issues as a community. Therefore, school violence is a problem encompassing students, faculties, parents, and others. Violence toward and attacks on teachers and staff impact not only the victims but also the overall school environment [5]. The involvement of parents and the community is crucial in addressing school violence. Consequently, school violence is a multifaceted issue that encompasses students, teachers, staff, parents, and the broader community, necessitating a comprehensive approach to prevention and intervention [6].

### 1.1. Negative Effects of School Violence

The negative effects of school violence are many. School violence is associated with behavioral, social, cognitive, and emotional well-being, absenteeism, and academic failure [7,8,9]. Its negative consequences include emotional abuse, difficulties forming attachments, depression, post-traumatic stress disorder, suicidal ideation, substance use, and public health [10,11,12,13,14,15,16]. In addition, as online users increase, cyberbullying in school violence increases, which includes verbal abuse, group violence, visual abuse, impersonation, account forgery, disclosure of private lives, sexual harassment, and cyberstalking using various online media (text/audio, messages, posts, photos, videos, etc.) [17,18,19,20].

### 1.2. Goal of the Study

This study explored prospective teachers’ perceptions of school violence and their role in addressing it. Until now, most research has been related to school violence concerning students and current teachers but there is a lack of research on prospective teachers. Based on this awareness of the problem, this study used Q methodology to delve into the role of teachers in addressing school violence, as viewed by prospective teachers in Korea. The study highlights the need to develop customized educational programs that incorporate prospective teachers’ views and suggests strategies for preventing and addressing school violence as essential components of teacher training.

The research questions (RQ) guiding this inquiry were as follows:

RQ1: From the perspective of prospective teachers, how do they perceive the teacher’s role in school violence?

RQ2: From a prospective teacher’s perspective, what are the characteristics of each role type of teacher regarding school violence?

## 2. Literature Review

### 2.1. Definitions and Elements of School Violence

Definitions of school violence can vary across countries and scholars. Regions like Europe, Canada, and Australia often recognize bullying as a form of school violence. These regions typically define bullying as repeated and persistent negative behaviors toward a student by one or more peers [21,22]. Bullying also occurs when a school-aged child uses unwanted aggressive behavior, exploiting a real or perceived power imbalance [23] to intentionally and repeatedly cause injury or discomfort to another person [24]. The commonalities of these definitions of school violence are relationships, aggression, intentionality, repetition, and a state of power imbalance [21,25].

In Korea, the legal definition of school violence is an act that causes physical, mental, or property damage through injury, assault, confinement, intimidation, kidnapping/luring, defamation/insult, extortion, coercion/forced errands, sexual violence, bullying, cyberviolence, etc., that occurs inside or outside of school [26]. In the process of revising this law, Korea expanded the scope of school violence to include acts committed ‘in school’ and those committed ‘outside of school.’ Similarly, the United States also considers bullying that occurs outside of school as school violence, as reflected in the recent Report on Indicators of School Crime and Safety: 2023. This report provides data and insights into the prevalence and impact of bullying beyond the school setting and highlights its broader societal implications [27].

### 2.2. Causes and Prevention of School Violence

Scholars point to the following as causes of school violence. First, the higher the incidence of domestic violence, the higher the incidence of school violence [28]. Second, a low sense of belonging within the school community, limited teacher–student interactions, and large class sizes have caused students to feel alienated or frustrated [29,30,31]. Moreover, studies on school-violence prevention reveal the importance of investing time and resources to prevent school violence, indicating that individual efficacy can increase if schools support students so they do not feel abandoned [32,33]. In addition, researchers found that school violence decreases through student and community participation, such as student mentoring, peer counseling, and activities related to policies and disciplinary issues [34,35,36,37].

### 2.3. Policy Efforts to Improve Korea’s Educational Environment and Solve School-Violence Problems

In Korea, the education system emphasizes rigorous academic achievement and places high expectations on students and teachers. In this pressure-filled environment, school-violence incidents have become an area of serious concern for educators, policymakers, and society. Accordingly, Korea has implemented various policies to address school violence based on domestic and international research findings. The suicide of a middle-school student in Daegu in 2011 elevated the seriousness of school violence. This tragedy shocked Korean society and strengthened public and government interest in addressing this issue [38,39].

In addition, due to this incident, the Office of Education conducts online school-violence surveys at the beginning of each semester [40]. This incident was the impetus for Korea passing the Act on the Prevention and Countermeasures of School Violence. This law aims to protect students’ human rights and foster them as sound members of society by protecting victimized students, guiding and educating the offending students, and mediating disputes between the victimized and the offending students.

A 2024 Ministry of Education survey on school violence reported a response rate of 2.1%, an increase of 0.2% from the previous year. Verbal violence constituted the highest proportion of incidents at 39.4%. The survey also identified group bullying, sexual violence, and extortion as serious social issues due to their year-over-year increase [41].

However, despite various measures such as enacting school-violence prevention programs and laws, the problem persists [42]. Accordingly, relevant departments jointly established “field-centered school violence countermeasures” to institutionally resolve it.

To become a teacher in Korea, one has to attend a teacher’s college and complete a separate course to obtain a teaching license. College students on this course are called prospective teachers. In order to foster the ability of prospective teachers to deal with problems related to school violence that may occur in school settings, subjects related to school violence have been added to the university curriculum. In addition, “School Violence Prevention and Student Understanding” was introduced as a required subject on the teacher’s license course [43].

## 3. Method

This study used Q methodology to assess prospective teachers’ perceptions of the role of teachers in dealing with school violence. Q methodology is a mixed-method approach that scientifically measures subjective meanings centered on personal perspectives, beliefs, assumptions, experiences, and social meanings [44,45]. It is an alternative approach to classify individuals’ subjective views and is a mixed-method approach that combines quantitative and qualitative methods [46,47]. It also allows participants to express their thoughts, acknowledging their previously unheard voices [48,49].

**Figure 1:**
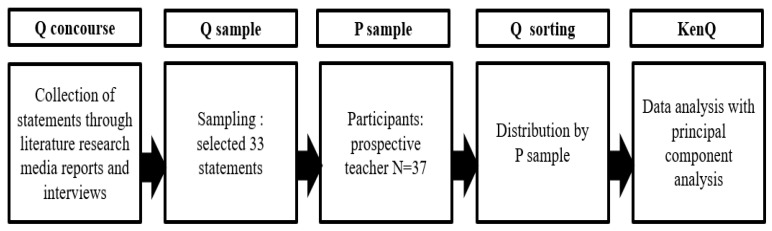
Research procedure.

Figure 1 illustrates this study’s research procedure. The process involved several steps. First, we composed statements by comprehensively reviewing relevant literature and newspaper articles. Then, we prepared a semi-structured questionnaire to understand the perceptions of teachers’ roles in dealing with school violence. The summary of these statements revealed various perspectives on teachers’ roles in dealing with school violence. Second, we sampled the final 33 statements. Third, we recruited prospective teachers for the study. Fourth, we engaged the teachers and conducted Q sorting to obtain their perspectives on teachers’ roles in dealing with school violence. Fifth, we ran the KenQ program on the population data to derive the results. We also obtained the prospective teachers’ written consent to participate in the study. In addition, the Institutional Review Board (IRB) designated by the Ministry of Health and Welfare reviewed and approved this study under IRB approval number DOIRB202304-08. Moreover, we gave sufficient consideration to the rights and privacy of the study’s participants.

### 3.1. Q Concourse

Q concourse refers to integrating statements by using conversations, interviews, and literature on a specific topic [50,51,52]. The main methods for collecting a Q concourse are literature reviews, preliminary data collections (subjective questions), and in-depth interviews to identify differences and commonalities in people’s perceptions, ensuring they capture a wide range of voices [53,54].

We constructed a Q concourse for this study by conducting a literature review, a survey, and focus-group interviews. First, we composed 52 sentences by referencing the literature, academic journals, and articles reported in the media using search terms related to school violence. Second, we asked prospective teachers for their thoughts on the role of teachers in dealing with school violence; teachers responded in writing. As a result, we received responses from 58 individuals and composed 98 statements. In addition, we supplemented the responses through focus-group interviews, for which we recruited six students. We conducted all interviews using Zoom 6.2.6. The interview subjects included three males and three females; three were third-grade students and three were fourth-grade. The interviewees’ academic majors included Korean, mathematics, geography, history, home economics, and physical education.

The interview questions comprised “What is school violence and what forms can it take”, “What should we do when school violence occurs”, “How can we support those who are victims of school violence”, and “What is the role of teachers in dealing with school violence?”. After obtaining the students’ consent, we recorded and transcribed their responses, creating 59 sentences. Through this process, we secured 209 items, ensuring the sentences were self-referential and relevant to the research topic. We wrote each statement with the participants in mind, aiming to express only one opinion per statement [45].

### 3.2. Q Sample

We organized the Q sample by comprehensively arranging the Q concourse and grouping the related statements. Then, we revised and rewrote unnecessary content such as similar or difficult-to-understand sentences in a self-referential manner [45,55] to ensure the Q sample accurately expressed each statement and that we could measure them scientifically [45]. To create the Q sample, researchers repeatedly read the 209 items and categorized sentences with similar meanings into 107 sentences.

We divided the Q sample into the following five categories: (1) school violence from the perspective of the perpetrator, (2) school violence from the perspective of the victim, (3) school violence from the perspective of bystanders, (4) school violence from the perspective of prevention, and (5) school violence from the perspective of ripple effects. After reviewing purposefulness, understandability, simplicity, etc., the final Q sample comprised 33 statements (Table 1). Finally, to increase the validity of the Q sample, two professors with experience in Q methodology research and school-violence prevention education verified the statements.

### 3.3. P Sample

A P sample uses a small group of participants selected to compare different topics [56,57,58]. The study investigated teachers’ roles in addressing school violence, selecting 37 respondents through purposive sampling based on their interest in the study. After we thoroughly explained the study’s purpose and procedures, the participants signed an informed consent form.

The P sample comprised 13 males and 24 females, of which 19 were in third-grade and 18 in fourth-grade. The participants came from various academic fields; two were studying home economics, two were studying drama, three were studying mathematics, three were studying English, three were studying Korean, three were studying Chinese, four were studying geography, six were studying chemistry, and 11 were studying physical education.

### 3.4. Q Sorting

Q sorting is the organizing of individuals’ subjective views, showing the extent to which the P sample agrees or disagrees with each statement on a given topic [56]. In other words, Q sorting involves modeling the P sample’s attitude toward a specific topic or situation; it is a process of reading statements and forcibly distributing them [45,55]. Thus, Q sorting involves ranking and classifying statements according to the P sample’s perspective.

**Figure 2:**
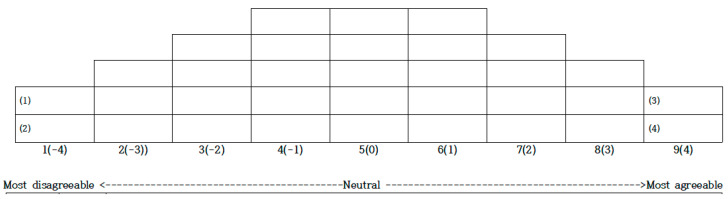
Data distribution diagram.

For this study, we gave 37 participants three sheets on Q sorting. First, we read the 33 statements multiple times to familiarize them with all the sentences. We also prepared Q cards for the participants to cut out and arrange. Second, the participants divided the Q cards into the following three categories: those with which they agreed, disagreed, or were neutral toward. Then, they placed the statements (Q cards) with which they most agreed on the right end of the Q sorting sheet (+4) and the statements they most disagreed with on the left end (−4). They sequentially arranged the Q cards and provided reasons for their selections. The researcher used a forced distribution method to adjust the arrangement to a normal distribution. This process took about 50 min. We assigned identification numbers (i.e., “P” numbers) to ensure participant anonymity (Figure 2).

### 3.5. Data Analysis

We used the computer program KENQ to analyze the collected data. In coding the Q sorting for each participant, we assigned −4 points to items rated as ‘strong disagreement’ and +4 points to the ‘strong agreement’ items. Then, we used a principal component factor analysis to derive eigenvalues. The research results focused on understanding and interpreting the characteristics of each type by considering sentences with an eigenvalue of 1.000 or more among the Z-scores [45]. In addition, we interpreted the characteristics of each type by examining the P sample’s reasons for agreeing or disagreeing with the items and the reasons behind the strongest agreements and disagreements observed during the Q sorting process. When interpreting the analysis results, we focused on the reasons behind the statements with which the P sample, with high factor weights, most strongly agreed or disagreed, as these high factor weights also indicated specific types.

## 4. Results

### 4.1. Type-by-Type P Sample and Weight Analysis

The Q classification results identified three perceptions regarding teachers’ roles in addressing school violence. As each type consisted of P samples with similar thoughts or opinions, we could distinguish each type’s perception characteristics (Table 2). The higher the factor weight for each type, the more representative the type. For example, P8 (10) represented Type 1, P9 (10.4129) represented Type 2, and P33 (9.6876) represented Type 3.

The eigenvalues of Types 1 to 3 were 21.7269, 2.7799, and 1.6298, respectively. The cumulative eigenvalues of the three types accounted for 71% of the variance, and each type explained 59% (Type 1), 8% (Type 2), and 4% (Type 3) of the variance (Table 3).

Correlation coefficients can explain the degree of similarity between types [45]. The correlation between Types 1 and 2 was 0.724, between Types 1 and 3 was 0.831, and between Types 2 and 3 was 0.709, indicating that the correlation between these types was relatively high (Table 4).

### 4.2. Characteristics of the Perception of Teachers’ Roles in Dealing with School Violence by Type

#### 4.2.1. Type 1: Prevention-Oriented Teacher

Type 1 emphasized the importance of school-violence preventive measures using teacher observation and guidance; 17 participants were this type. Type 1 strongly agreed with the importance of ‘teacher’s careful observation and continuous guidance’ (Q10; z = 1.681) to prevent school violence in advance. They emphasized the need for teachers to observe and guide students’ behaviors before school violence occurs. They strongly agreed with the statement “We need to change bystanders into defenders” (Q20; z = 1.413). They most disagreed with “It is natural to get hit if you don’t listen” (Q22; z = −1.879) and “There is a good reason to be bullied or harassed” (Q9; z = −1.92) (Table 5).

P8 (10), with a high factor weight, stated that “School violence is a problem that mostly occurs between students, but teachers’ careful observation and guidance can prevent school violence. However, students need to act defensively in places where teachers are not visible”. P29 (8.8941) and P10 (8.5099) stated that “Victims or witnesses of school violence cannot report it themselves because of fear. They do not seek out teachers because of retaliation. Therefore, teachers should discover it first and report and punish them”. In addition, P15 (7.5749) and P6 (6.7493) stated the following:

“School violence occurs without me knowing. The related punishment laws and systems are already sufficiently in place. Also, the perpetrators do not empathize with what they are doing wrong. Therefore, it is necessary to improve the relationship with teachers so that school violence does not spread like mold. In other words, school violence will decrease if the relationship between students and teachers improves”.

Prospective teachers belonging to this type held the view that teachers should discover school violence in advance and actively intervene in school-violence situations. In particular, the difference from other types was that teachers should first discover and deal with situations where victims could not report due to fear of retaliation. They also thought that improving the relationship between teachers and students was essential to prevent school violence. They argued that improving the relationship between teachers and students could be an important defense to prevent school violence from secretly spreading.

They also recognized that teachers’ continuous interest and observations were important factors for preventing school violence in advance because they provided psychological stability to students. However, Type 1 showed a cautious stance on punishment for school violence. In Q22 and Q9, they argued that “Problem-solving through dialogue is necessary rather than punishment for violence” and they preferred an approach that “analyzes the cause of violence and makes the perpetrator understand their behavior”.

The characteristic of Type 1 was that the key to preventing school violence was the teacher’s observation and guidance before the problem occurred. Teachers needed to observe and guide within the school and create an environment where victims and witnesses could report safely. This type suggested practical ways for all school community members to cooperate to prevent violence and emphasized that teachers, students, and parents must respond together.

#### 4.2.2. Type 2: Reality-Avoidant Teacher

Type 2 emphasized avoiding this situation as much as possible to prevent potential harm from getting involved in school violence. Our study identified 13 participants in this type. Type 2 was aware of school-violence situations and knew how to deal with them but they believed that school violence would never disappear, so they tried to avoid it in reality (Table 6). Type 2 most agreed with the statements “School violence is hard to eliminate” (Q11; z = 1.613), “School violence leaves trauma to the victimized students” (Q30; z = 1.488), and “School violence is much more common than is revealed” (Q18; z = 1.441). They were the type who knew that school violence had occurred but were afraid their involvement would cause them harm. Therefore, they found it safer to avoid school-violence situations.

P9 (10.4129), with a high factor weight, showed an evasive attitude. It stated the following:

“School violence that occurs online and offline is not limited by time and space. It is impossible to eliminate school violence. Also, if you try to solve school violence, you are afraid of retaliation against you or the destruction of your interpersonal relationships, so you are reluctant to step forward actively”.

Further, P30 (9.8941) recognized the following statement:

“School violence lowers your self-esteem and becomes a big trauma that is hard to erase. Also, bullying by someone can be hurtful and scary to the victim, but some students feel superiority or satisfaction by bullying others, so it is hard to eliminate”.

In addition, P20 (7.8027) stated that “The level of school violence is getting stronger, and its frequency is increasing. Therefore, teachers have limitations in preventing it. If teachers fail to solve the problem, they fear becoming another victim, so it is better to avoid it”.

The characteristic of Type 2 was that teachers were aware of the dangers of school violence but they did not actively respond, choosing instead to avoid it because they thought it was dangerous for them to step forward to solve school violence. They believed that it was impossible to eliminate school violence but were realistic at finding ways to minimize the situation or reduce trauma rather than solve the problem. They also believed that teachers’ helplessness and fear could be a major obstacle to solving the problem of school violence. Therefore, they emphasized that teachers, students, and parents within the school community should change their attitudes toward accepting the problem of school violence.

#### 4.2.3. Type 3: Legal-Regulation-Oriented Teacher

Type 3 emphasized the need to provide clear rules and norms related to school violence and prevent school violence through education for students and teachers. Seven participants belonged to this type. Type 3 strongly recognized the need to strengthen rules and legal devices to prevent and resolve school violence (Table 7). They strongly agreed that “School violence can cause trauma to victims” (Q30; z = 1.778) and highlighted that schools should prevent and eradicate school violence by emphasizing specific rules and legal responsibilities such as “operating a school violence prevention program” (Q31; z = 1.627).

P33 (9.6876), with the highest factor weight, argued for “strengthening punishment rules and expanding school violence prevention programs to create a violence-free school”. P3 (6.5576) stated that “victims can experience continuous trauma even after incident resolution, and this can affect them even after they become adults, so legal protection measures should be in place”. In addition, P28 (2.6123) criticized the situation where “the perpetrator avoids legal responsibility, and the victim has to fight loneliness alone, as seen in dramas and movies”. They emphasized that school-violence perpetrators should be held strongly and legally accountable. They believed that school violence could spread further if there were no clear rules and legal procedures, and that schools could strengthen prevention and responsibility by educating students and teachers about these rules in advance.

The characteristic of Type 3 was that school violence would increase if rules or norms were lowered or eliminated. In addition, as individuals could exploit rule or norm insufficiencies to cause other school violence, presenting clear rules and providing prior education to students and teachers could help to prevent school violence.

## 5. Discussion

This study used Q methodology to examine how prospective teachers perceive the role of teachers in addressing school violence, identifying and analyzing different perception types and their characteristics. The study aimed to provide basic data for prospective teachers to develop their capabilities as experts to properly manage school violence. As a result of the study, we classified the roles of teachers in dealing with school violence from the perspective of prospective teachers into prevention-oriented teachers (Type 1), reality-avoiding teachers (Type 2), and legal-regulation-oriented teachers (Type 3).

### 5.1. Type 1: Prevention-Oriented Teachers

Prevention-oriented teachers (Type 1) represented teachers who discovered school violence in advance and actively intervened. They believed that the trust relationship between teachers and students effectively prevented school violence. This finding was consistent with previous studies in that teachers’ meticulous attention and students’ active interventions contribute to creating a school-violence prevention culture [59]. It was also consistent with research results that students are more likely to ask teachers for help with school violence when they respect and empathize with students [59,60]. This approach is important in reducing school violence by increasing students’ self-efficacy [16,32,33]. It is also consistent with research results indicating that school violence will decrease as community participation such as student mentoring and peer counseling becomes more active in prevention [34,35].

Korea is conducting school-violence prevention education for students, teachers, and parents through the Eoullim Program, and overseas, research results show that the Kiva Koulu, Olweus Program, Second Step, and other initiatives effectively prevent school violence [61,62,63,64,65]. In the teacher training course in Korea, prospective teachers acquire the ability to deal with school violence through the “School Violence Prevention and Student Understanding” [66]. Building trust between teachers and students and strengthening preventive approaches through these programs and curricula is important. In addition, policies that offer emotional support and psychological stability for students and teachers are essential.

### 5.2. Type 2: Reality-Avoiding Teachers

Type 2, avoidant teachers, showed an attitude that it had nothing to do with them, even when they witnessed school violence as bystanders. This type of teacher recognized the seriousness of school violence but tended to avoid it out of fear of being harmed. This avoidant attitude was consistent with research associated with depression and suicide risk [67]. This avoidance did not solve the problem but demonstrated submission and could encourage further victimization [68]. Type 2 pointed out that we should view school violence as a problem of the entire group, not an individual problem. They claimed that educational programs that transformed bystanders into defenders, such as the “Say Something Anonymous Reporting System”, effectively improved avoidant attitudes [69,70,71]. Type 2 also claimed that it was necessary to create an environment in which teachers could actively respond to school violence. To this end, these individuals emphasized the need for a safe reporting environment and programs that protected teachers.

### 5.3. Type 3: Legal-Regulation-Oriented Teachers

Type 3, the legal-regulation-oriented type, argued for strengthening of the rules and norms to resolve school violence. This type of teacher wanted more stringent regulations applied to perpetrators and insisted that specific behavior correction was necessary rather than simple reprimands [72]. In particular, they emphasized recognizing mental damage from things such as verbal abuse or group bullying as part of school violence, and that clear punishment regulations could reduce the damage. Korea has strengthened its school-violence prevention through rules such as the immediate suspension of attendance for perpetrators, the mandatory recording of disciplinary matters in school records, strengthening guidance for preventing school violence on campus, strengthening the description of the character in school records, and utilizing admissions-officer evaluation factors; the role of the deliberation committee is important in this regard [35,73].

### 5.4. Significance and Policy Implications

This study is significant as it analyzed the role of teachers in addressing school violence from teachers’ perspectives, suggesting customized educational programs for each identified type.

This study revealed the following policy implications. First, schools need to construct a curriculum on school-violence prevention and response measures, shifting from a theory-centered approach to one linked with practical field cases. This curriculum will help teachers to develop the ability to appropriately deal with school violence in the early stages. Second, schools should expand mental-health and character-education programs for teachers to support them to develop conflict-resolution skills and protect their authority. These programs are necessary because teachers’ mental health is an important factor in solving school-violence problems beyond student guidance. Third, students and teachers should be at the center of solving the problem of school violence, and it is necessary to minimize excessive interference from parents and focus on reconciliation and growth among students. This approach requires policies that focus on the essence of resolving school-violence issues to ensure smooth communication between parents, teachers, and students and prevent them from escalating into legal disputes.

## 6. Limitations

This study focused on distinguishing and identifying the roles of teachers in dealing with school violence from the viewpoint of Korean prospective teachers but there were several limitations. First, due to the nature of Q methodology, the sample size was small, thus limiting the results’ generalizability. We recommend follow-up studies with more participants and exploring a broader perspective with prospective teachers from various regions and backgrounds. Second, as the study reflected the cultural context of education and school violence in Korea, the results may differ when conducting similar studies in other cultures or countries. We recommend conducting international studies comparing how prospective teachers or teachers in other countries perceive school violence. Finally, there was a lack of in-depth analyses of how psychological and emotional factors affected the perception of school violence. Thus, we need to research prospective teachers’ perceptions by considering various psychological factors such as stress levels and psychological exhaustion.

## 7. Conclusions

This study categorized the roles of teachers in dealing with school violence from the perspective of prospective teachers into three categories and suggested policy implications. Each type plays an important role in solving school-violence problems, and the effectiveness of school-violence prevention can vary depending on the response method for each type.

In conclusion, preventing school violence requires collaboration among various stakeholders, including parents, youth leaders, community residents, and teachers. In particular, teachers must prevent dangerous situations in advance based on a correct understanding of school violence and actively intervene in post-event guidance. To this end, we must establish an education and support system that enables prospective teachers to understand and respond to school-violence issues. Schools should build and strengthen a safe reporting system and teacher-protection programs to create an environment where teachers can more actively intervene in school violence. In addition, it is necessary to expand school-violence prevention programs and educate students and teachers about their legal responsibilities and rights.

## Figures and Tables

**Table 1:** Q sample.

No.	Statement
Q1	Hitting or teasing a friend as a joke is not school violence.
Q2	Calling someone by an unwanted nickname is also school violence.
Q3	The perpetrator must know of the victim’s suffering.
Q4	Each class needs helpers to prevent school violence.
Q5	School violence corrupts school life like mold.
Q6	When I think of school violence, I feel guilty.
Q7	School violence requires active reporting.
Q8	It’s natural for a child who is cocky to be bullied or harassed.
Q9	All children who are bullied or bully have a reason.
Q10	We need teachers’ careful observation and continuous guidance to prevent school violence.
Q11	I think school violence is hard to eliminate.
Q12	School violence alienates students.
Q13	You should help a friend bullied at school, but it is not easy.
Q14	Emotional comfort is needed to heal school violence.
Q15	If another friend hits me, I have to hit him too.
Q16	School violence can lead to retaliatory violence.
Q17	Rules for dealing with perpetrators should be clearly defined and enforced.
Q18	I think school violence is much more than schools reveal.
Q19	School violence is when friends fight for no reason.
Q20	To prevent school violence, we must transform bystanders into defenders.
Q21	Psychotherapy is needed to treat school violence.
Q22	Those who misbehave deserve a beating.
Q23	The perpetrator must make a sincere apology to the victim.
Q24	The School Violence Committee must be operated fairly.
Q25	School violence makes school a place you don’t want to go to.
Q26	If I witnessed school violence, I’d avoid it.
Q27	It is school violence that causes sexual shame.
Q28	School violence occurs because there is no empathy for the other person.
Q29	School violence has a bad effect on improving relationships.
Q30	School violence traumatizes the victim.
Q31	Schools should operate violence prevention programs.
Q32	School violence lowers self-esteem.
Q33	School violence is an act of teasing or harassing another person by taking advantage of their weakness.

**Table 2:** P samples and factor weights.

Type	No.	Factor Weight	Sex	Grade	Teacher Certification Subjects
Type 1 (n = 17)	P8	10	M	3	Mathematics
	P29	8.8941	F	3	Korean Language
	P10	8.5099	F	4	Chinese
	P24	7.9368	F	3	Geography
	P15	7.5749	M	3	Physical Education
	P6	6.7493	F	3	Home Economics
	P2	6.1432	F	4	Mathematics
	P7	6.1169	F	4	Physical Education
	P13	5.7304	F	4	Physical Education
	P4	4.5622	M	4	Physical Education
	P14	4.0695	F	4	Chemistry
	P18	4.0695	F	4	Chemistry
	P16	3.8755	M	3	Korean Language
	P17	3.8538	M	3	Physical Education
	P1	3.5011	M	4	Physical Education
	P11	3.2466	F	4	Physical Education
	P12	3.1879	M	3	Physical Education
Type 2 (n = 13)	P9	10.4129	M	3	Physical Education
	P23	10.0298	M	3	Physical Education
	P27	9.8941	F	3	Chemistry
	P25	8.7448	M	3	Geography
	P35	7.8027	F	3	Geography
	P20	6.9663	M	3	Mathematics
	P31	6.1406	F	3	Theatre and Film
	P21	5.6275	F	3	Chemistry
	P32	4.2937	F	4	Geography
	P36	4.0049	F	3	English
	P19	3.8796	F	4	English
	P5	3.4512	F	4	Chemistry
	P30	3.1127	F	4	Chinese
Type 3 (n = 7)	P33	9.6876	F	4	Chemistry
	P3	6.5576	F	4	Chinese
	P26	5.2365	F	4	Home Economics
	P22	4.7615	M	4	Physical Education
	P34	4.4130	F	4	Theatre and Film
	P37	4.3920	M	3	Korean Language
	P28	2.6123	F	3	English

**Table 3:** Variance analysis.

Content/Type	I	II	III
Eigenvalues	21.7269	2.7799	1.6298
% Explained Variance	59	8	4
Cumulative % Explained Variance	59	67	71

**Table 4:** Variance analysis.

Type	I	II	III
I	1.000		
II	0.724	1.000	
III	0.831	0.709	1.000

**Table 5:** Type 1 statements and z-scores (higher than 1.00).

No.	Q Statement	Z-Score
Q10	We need teachers’ careful observation and continuous guidance to prevent school violence.	1.681
Q20	To prevent school violence, we must transform bystanders into defenders.	1.413
Q23	The perpetrator must make a sincere apology to the victim.	1.155
Q30	School violence traumatizes the victim.	1.151
Q6	When I think of school violence, I feel guilty.	−1.066
Q15	If another friend hits me, I have to hit him, too.	−1.292
Q26	If I witnessed school violence, I’d avoid it.	−1.362
Q8	It’s natural for a child who is cocky to be bullied or harassed.	−1.639
Q1	Hitting or teasing a friend as a joke is not school violence.	−1.701
Q9	All children who are bullied or bully have a reason.	−1.726
Q22	Those who misbehave deserve a beating.	−1.879

**Table 6:** Type 2 statements and z-scores (higher than 1.00).

No.	Q Statement	Z-Score
Q11	I think school violence is hard to eliminate.	1.613
Q30	School violence traumatizes the victim.	1.488
Q18	I think school violence is much more than schools reveal.	1.441
Q25	School violence makes school a place you don’t want to go to.	1.276
Q13	You should help a friend bullied at school, but it is not easy.	1.247
Q26	If I witnessed school violence, I’d avoid it.	−1.171
Q1	Hitting or teasing a friend as a joke is not school violence.	−1.305
Q15	If another friend hits me, I have to hit him, too.	−1.395
Q8	It’s natural for a child who is cocky to be bullied or harassed.	−1.565
Q9	All children who are bullied or bully have a reason.	−1.745
Q22	Those who misbehave deserve a beating.	−1.967

**Table 7:** Statements and z-scores for Type 3 (higher than 1.00).

No.	Q Statement	Z-Score
Q30	School violence traumatizes the victim.	1.778
Q31	Schools should operate violence prevention programs.	1.627
Q33	School violence is an act of teasing or harassing another person by taking advantage of their weakness.	1.147
Q7	School violence requires active reporting.	1.061
Q27	It is school violence that causes sexual shame.	1.005
Q6	When I think of school violence, I feel guilty.	−1.087
Q26	If I witnessed school violence, I’d avoid it.	−1.155
Q1	Hitting or teasing a friend as a joke is not school violence.	−1.359
Q15	If another friend hits me, I have to hit him, too.	−1.427
Q22	Those who misbehave deserve a beating.	−1.661
Q9	All children who are bullied or bully have a reason.	−1.995
Q8	It’s natural for a child who is cocky to be bullied or harassed.	−2.125

## Data Availability

The datasets generated and/or analyzed during the current study are available from the corresponding author upon reasonable request.
